# Plant-Derived Polyphenols in Osteoarthritis: Mechanisms, Delivery Systems, and Therapeutic Synergy

**DOI:** 10.3390/life16101653

**Published:** 2026-09-30

**Authors:** Mohd Farhan

**Affiliations:** Department of Chemistry, College of Science, King Faisal University, Al Ahsa, Saudi Arabia; mfarhan@kfu.edu.sa

**Keywords:** osteoarthritis, polyphenols, synovial inflammation, advanced drug delivery systems, combination therapy

## Abstract

Osteoarthritis (OA) is a common, disabling joint disease characterized by complex inflammatory, oxidative, and catabolic cascades that culminate in progressive cartilage degradation, subchondral bone remodeling, and synovitis. Standard pharmacological treatments such as nonsteroidal anti-inflammatory drugs (NSAIDs) provide symptomatic relief of pain but do not prevent the progression of the disease and are often associated with serious gastrointestinal, renal and cardiovascular side effects following long-term use. Epigallocatechin-3-gallate (EGCG), resveratrol, curcumin, quercetin, luteolin and genistein are naturally occurring polyphenols which have been identified as promising disease-modifying osteoarthritis drugs (DMOADs). Mechanistically, these phytochemicals suppress key pro-inflammatory signaling pathways (NF-κB, MAPK), downregulate matrix-degrading enzymes (MMP-13, ADAMTS-5), mitigate reactive oxygen species (ROS), and attenuate chondrocyte apoptosis and senescence. Polyphenols have been shown in pre-clinical and clinical trials to reduce joint pain and preserve cartilage integrity. They also have a synergistic effect with conventional drugs, resulting in lower doses and reduced toxicity. However, their clinical translation has been hampered by poor oral bioavailability, rapid Phase II metabolism, and short intra-articular retention. Advanced drug-delivery systems, such as nanoparticles, liposomes, and sustained-release hydrogels, are essential to overcome these pharmacokinetic bottlenecks and realize their full therapeutic potential in OA management.

## 1. Introduction

Osteoarthritis (OA) represents the most prevalent type of arthritis worldwide, acting as a leading cause of disability among older adults. While its prominence as an aging-associated disorder is well established, its etiology encompasses multiple other risk factors, including genetic predisposition, gender, previous trauma, and metabolic syndrome. Previously considered merely a “wear and tear” condition defined by cartilage depletion, OA is now understood as a highly complex pathology involving progressive articular cartilage destruction, subchondral bone remodeling, and synovial inflammation. The primary clinical manifestations—pain and restricted mobility—frequently force OA patients into a sedentary lifestyle, which consequently elevates their risk for cardiovascular disease and premature mortality [1].

**Figure 1:**
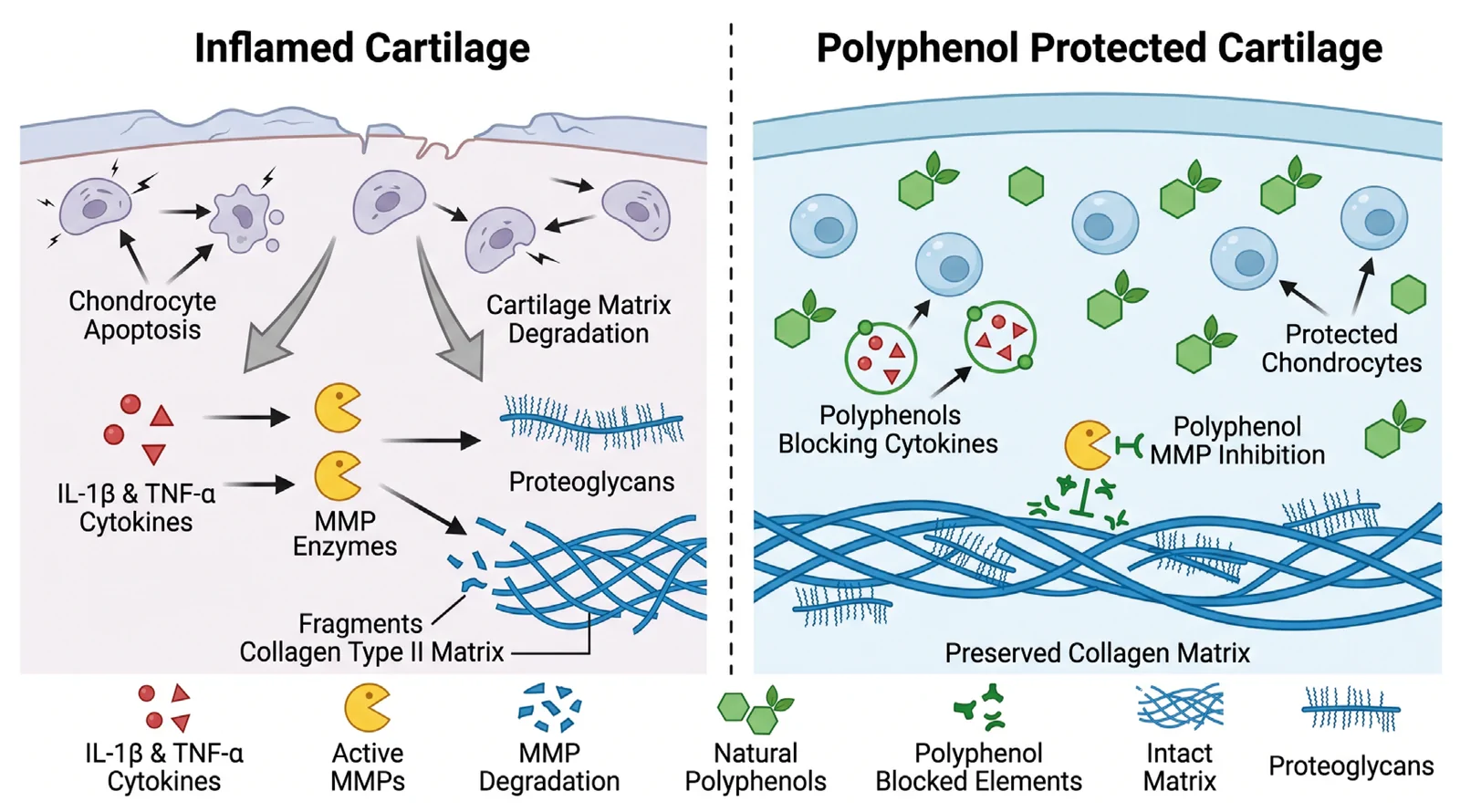
Mechanisms of polyphenols protection of cartilage. Illustration of Osteoarthritic Inflammation and Degradation (**left**) vs. Polyphenol-Mediated Matrix Preservation (**right**). It clearly demonstrates cytokine inhibition, matrix degradation, and chondrocyte protection.

Although OA is not classified as a classic autoimmune inflammatory disorder such as rheumatoid arthritis, inflammation remains a crucial driver of its pathogenesis. Localized inflammatory events in the synovial lining, termed synovitis, initiate chronic responses that aggravate clinical symptoms and hasten structural joint deterioration. These mechanisms include the secretion of pro-inflammatory cytokines, Tumour necrosis factor-alpha (TNF-α) and Interleukin-1 beta (IL-1β), and the activation of signaling cascades, such as nuclear factor-kappa B (NF-κB) (Figure 1) [2,3]. Furthermore, the subchondral bone serves as a reservoir of inflammatory mediators that contribute to OA-related pain and the destruction of the deeper layers of cartilage [4]. OA is also associated with low-grade systemic inflammation in addition to localized joint inflammation. Importantly, patients with increased IL-1β gene expression in peripheral blood leukocytes have greater pain, reduced physical function, and a higher likelihood of radiographic progression in OA [5]. This low-grade systemic inflammatory state may also favor the development of comorbidities, including stroke, Alzheimer’s disease, and myocardial infarction [4].

Currently, there is no cure for OA. The main goals of clinical management are pain relief and slowing disease progression [6]. Standard therapeutic measures include intra-articular injections of agents such as dexamethasone and hyaluronic acid, as well as oral administration of non-steroidal anti-inflammatory drugs (NSAIDs) [7]. However, long-term use of these conventional medications for treatment of OA symptoms can cause significant damage to different organ systems, especially in cardiovascular, gastrointestinal, and renal health [8]. There is thus a critical clinical need to develop new therapeutic regimens that offer long-term relief of OA symptoms with minimal adverse effects.

Polyphenols of natural origin have great therapeutic potential due to their proven tissue-protective, antioxidant, and anti-inflammatory properties [7]. These naturally occurring polyphenols can modulate the metabolic and inflammatory cascades underlying OA, relieve clinical symptoms, and potentially slow the disease’s pathological progression [9]. As such, exploring the therapeutic potential of these botanicals in OA management responds to growing public interest in natural health alternatives and offers novel approaches to treating this increasingly common age-related condition.

Symptomatic management is still the mainstay of conventional pharmacotherapy for osteoarthritis (OA). Non-steroidal anti-inflammatory drugs (NSAIDs) and paracetamol (acetaminophen) are the most frequently prescribed first- and second-line agents, respectively. However, their utility in long term management is limited by well-known adverse effects of NSAIDs such as cardiovascular, gastrointestinal and renal toxicities and hepatic toxicity with chronic use of paracetamol. A systematic network meta-analysis by da Costa et al. [10] evaluating the efficacy and safety of NSAIDs, opioids, and paracetamol in knee and hip OA found that paracetamol provides clinically negligible pain relief irrespective of dose, while the best performing NSAIDs, etoricoxib and high-dose diclofenac, have significant safety trade-offs in the long-term [10]. This clinical landmark provides an imperative rationale to investigate natural polyphenols not necessarily as total replacements but as important NSAID-sparing or adjunctive therapies to reduce pharmaceutical side effects while maintaining systemic safety. The main aim of this review is to provide an overview of the use of natural plant polyphenols in modulating pain and the progressive pathology of osteoarthritis.

## 2. Osteoarthritis Physiopathology

Osteoarthritis (OA) was historically characterized as a degenerative, passive process governed primarily by mechanical “wear and tear” that resulted in the erosion of articular cartilage. However, modern molecular biology, advanced imaging, and systemic biomarker research have fundamentally redefined OA as a complex, active, and highly regulated pathological process affecting the joint as a whole organ [11]. The disease involves structural and functional failure across all joint tissues, including articular cartilage, subchondral bone, synovium, menisci, intra-articular ligaments, periarticular muscles, and the infrapatellar fat pad [12].

The pathogenesis of OA is driven by an imbalance between anabolic and catabolic cellular processes, initiated by a combination of mechanical stress, systemic metabolic dysfunction, low-grade chronic inflammation, and genetic predisposition [13]. Rather than being a single pathological entity, OA represents a converging phenotype of multiple distinct pathophysiological endotypes—ranging from post-traumatic and age-related endotypes to metabolic and inflammatory sub-phenotypes [14]. Central to this pathophysiological continuum is the breakdown of homeostatic crosstalk between joint tissues, creating a self-perpetuating degenerative cascade.

### 2.1. Articular Cartilage Degeneration and Chondrocyte Phenotypic Switch

Articular cartilage is a tissue that is avascular, alymphatic, and aneural, with a large extracellular matrix (ECM) maintained by a sparse population of specialized cells called chondrocytes [15]. In physiological conditions, chondrocytes maintain structural integrity by finely balancing the synthesis of ECM macromolecules, mainly type II collagen and aggrecan, with their enzymatic degradation. In OA, this homeostatic state is disrupted by a profound phenotypic shift in chondrocytes induced by mechanical overload or metabolic damage [16].

### 2.2. Extracellular Matrix Breakdown

The loss of structural integrity begins with the degradation of aggrecan, a large aggregating proteoglycan that provides cartilage with compressive resistance through osmotic hydration [17]. The enzymes mainly responsible for aggrecan depletion are members of the ADAMTS (A Disintegrin and Metalloproteinase with Thrombospondin Motifs) family, namely ADAMTS-4 and ADAMTS-5 [18].

After proteoglycan loss, matrix metalloproteinases (MMPs) irreversibly degrade the type II collagen fibrillar meshwork that confers tensile strength to cartilage. The major catabolic enzyme in this process is MMP-13 (collagenase-3), which shows much higher substrate specificity for type II collagen than MMP-1 or MMP-8 [19]. The breakdown of collagen leads to the opening of the matrix structure, followed by irreversible swelling, loss of mechanical elasticity, and exposure of pericellular matrix components to further proteolytic degradation.

### 2.3. Chondrocyte Hypertrophy, Senescence, and Apoptosis

Chondrocytes respond to changes in the microenvironment by entering an aberrant differentiation program reminiscent of endochondral ossification [20]. Runx2 (Runt-related transcription factor 2) is the master transcription factor controlling osteoarthritic chondrocytes and induces a hypertrophic phenotype with upregulation of markers including type X collagen (COL10A1), alkaline phosphatase (ALP), and osteocalcin [21]. Hypertrophic chondrocytes actively promote matrix calcification and secrete the vascular endothelial growth factor (VEGF), which promotes vascular invasion from the underlying bone.

In parallel, cellular stress induces chondrocyte senescence. Senescent chondrocytes express increased levels of p16INK4a and p53/p21 and become permanently cell-cycle arrested, secreting a proinflammatory, pro-catabolic secretome known as the Senescence-Associated Secretory Phenotype (SASP) [22]. Senescence-associated secretory phenotype (SASP) components, including interleukin-1 beta (IL-1β), interleukin-6 (IL-6), and MMPs, promote senescence of neighboring healthy cells through paracrine signaling. Finally, high levels of oxidative stress and chronic catabolic signaling induce chondrocyte apoptosis and necroptosis, and result in depletion of the cell population necessary for matrix repair [22].

### 2.4. Synovial Inflammation and Molecular Cross-Talk

**Figure 2:**
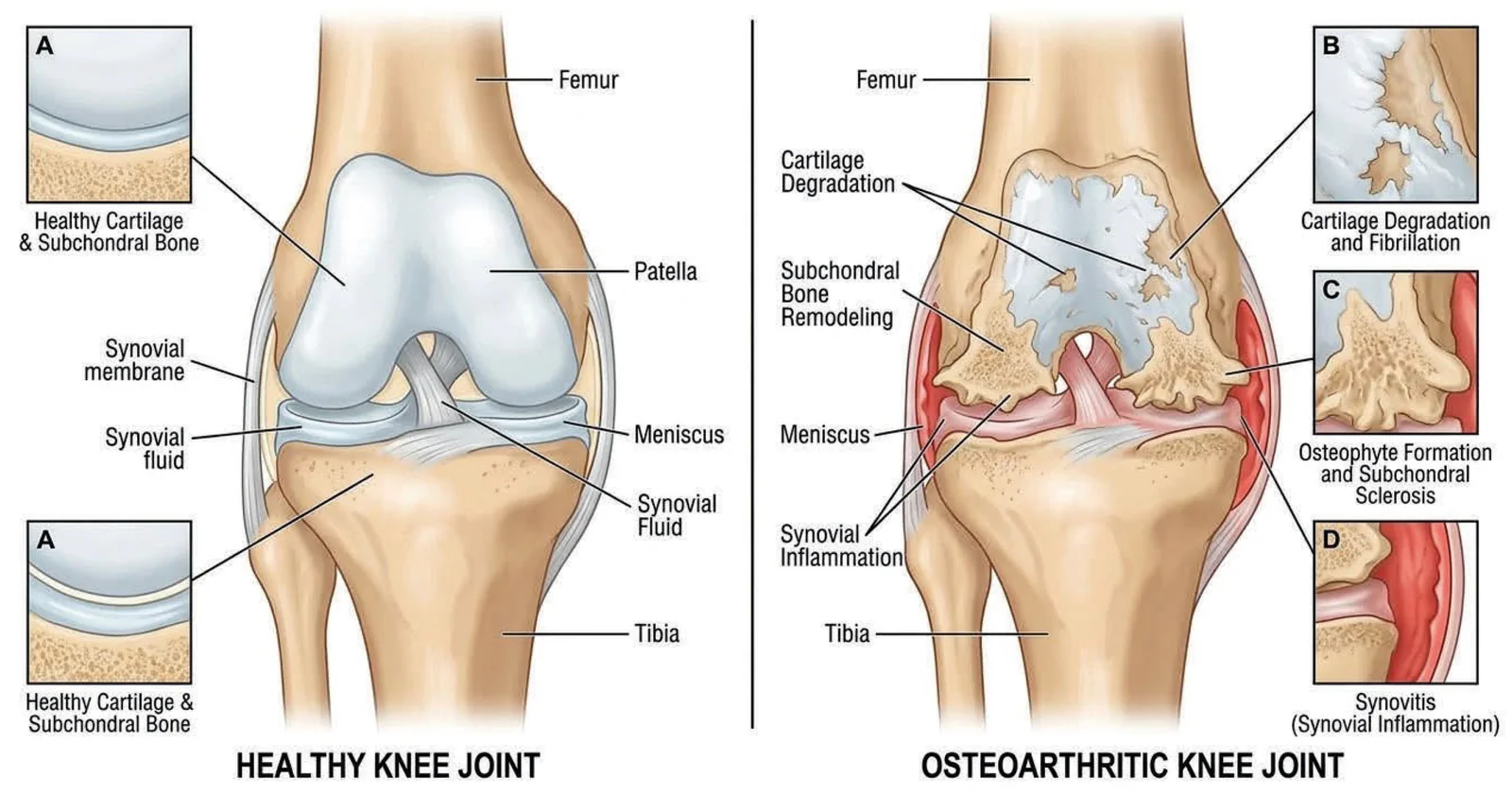
The image compares a healthy knee and an osteoarthritic knee. It shows the disease with progressive loss of articular cartilage, remodeling of subchondral bone, and focal synovial inflammation. These structural changes indicate a disruption of homeostatic crosstalk in joint tissue.

OA is classically described as a non-inflammatory arthropathy to differentiate it from autoimmune diseases such as rheumatoid arthritis, but low-grade, subclinical synovial inflammation (synovitis) is now recognized as a major driver of disease progression and pain [23]. Synovial hypervascularity, hyperplasia of the synovial lining, and infiltration of mononuclear immune cells—predominantly macrophages—are hallmark features of osteoarthritic synovium (Figure 2) [24].

### 2.5. Damage-Associated Molecular Patterns (DAMPs) and TLR Signaling

Degradation products released from the failing cartilage matrix act as endogenous danger signals, known as Damage-Associated Molecular Patterns (DAMPs) [25]. Fibrillar fragments of fibronectin, low-molecular-weight hyaluronan, tenascin-C, and S100 proteins escape into the synovial fluid. These DAMPs bind to pattern recognition receptors, particularly Toll-like Receptor 2 (TLR-2) and Toll-like Receptor 4 (TLR-4), expressed on synovial macrophages and synoviocytes [26].

Activation of TLR signaling recruits the adaptor protein MyD88, triggering downstream activation of Nuclear Factor-kappa B (NF-κB) and Mitogen-Activated Protein Kinase (MAPK) cascades [27]. This signaling hub drives the transcription of key proinflammatory cytokines:

- Interleukin-1 beta (IL-1β) and Tumor Necrosis Factor-alpha (TNF-α): Potent suppression of proteoglycan and collagen synthesis accompanied by robust upregulation of MMP-1, MMP-3, MMP-13, ADAMTS-4, and ADAMTS-5 [28].
- Interleukin-6 (IL-6): Synergizes with IL-1β to amplify osteoclastogenesis and synovial hyperplasia [29].
- Prostaglandin E2 (PGE2) and Nitric Oxide (NO): Synthesized via inducible cyclooxygenase-2 (COX-2) and inducible nitric oxide synthase (iNOS), respectively; these mediators inhibit matrix synthesis, promote cell death, and sensitize peripheral nociceptive neurons [30].

### 2.6. Metabolic Dysfunction and Obesity-Associated Osteoarthritis

Metabolic dysfunction, adipokine secretion, and low-grade systemic inflammation play a crucial role in obesity-associated knee OA; therefore, targeted metabolic and weight-loss interventions have emerged as potent disease-modifying strategies. In this context, the randomised, double-blind STEP 9 clinical trial by Bliddal et al. [31] underscored this axis by demonstrating that once-weekly subcutaneous semaglutide (2.4 mg) in individuals with obesity and knee OA resulted in profound weight loss associated with clinically meaningful reductions in WOMAC pain scores and physical function impairment compared with placebo [31]. In addition to the above clinical trial outcomes, real-world observational data from a 6-month prospective cohort study by Conforti et al. demonstrated that semaglutide treatment resulted in significant improvements in pain, WOMAC subscales, and key systemic inflammatory biomarkers (hs-CRP and TNF-α) in patients with knee OA [32]. The randomised controlled design of the STEP 9 trial provides definitive evidence of efficacy, and the real-world cohort by Conforti et al. provides valuable, pragmatic insight into clinical outcomes, albeit with the inherent limitations of observational design. Collectively, these metabolic therapies reinforce the therapeutic principle that modulation of systemic low-grade inflammation and metabolic stress can profoundly impact OA, providing a clear conceptual link to plant-derived polyphenols that target similar systemic inflammatory and metabolic pathways.

## 3. Polyphenols Targeting Osteoarthritis

**Figure 3:**
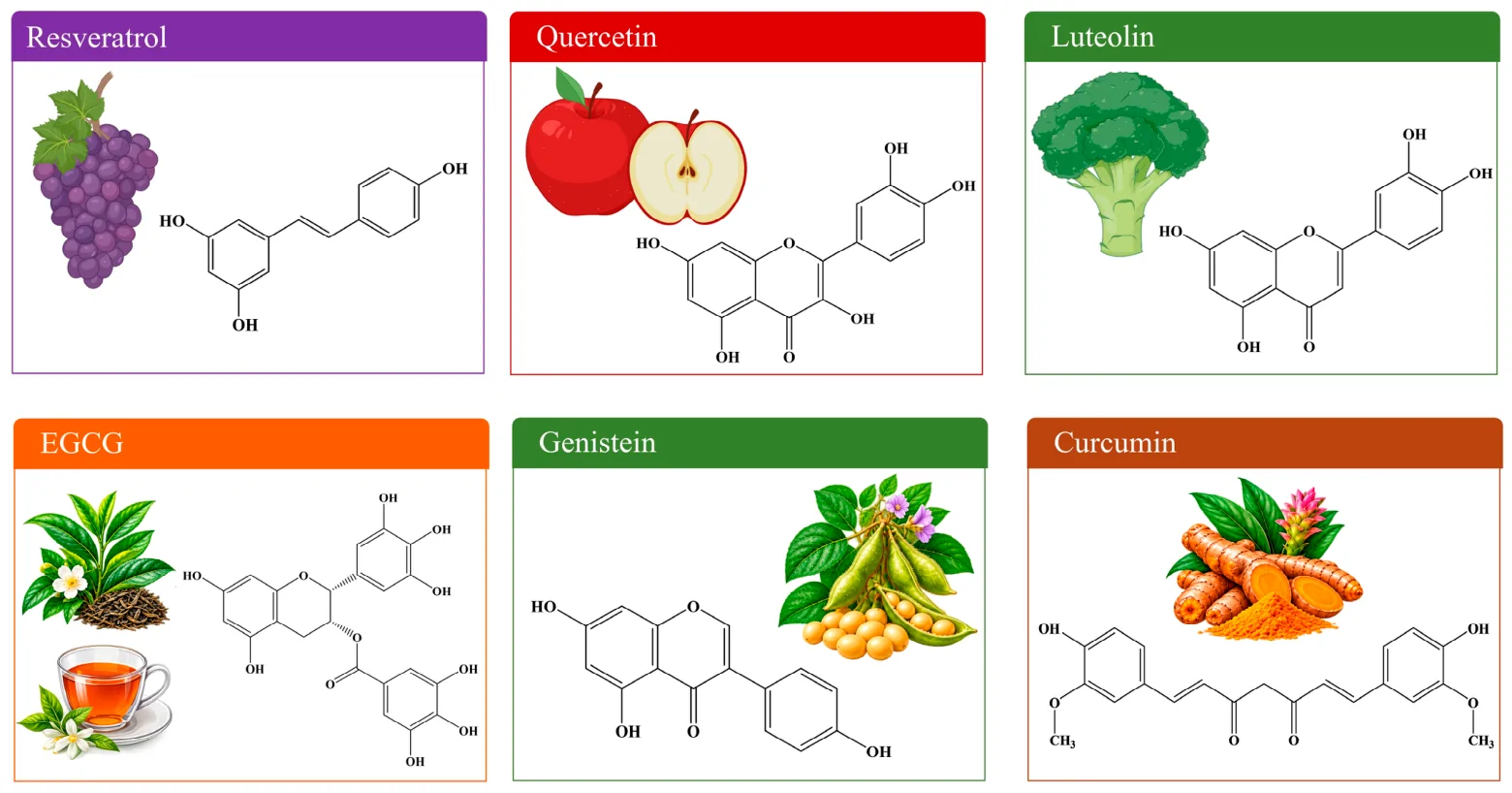
This composition highlights the diverse natural origins of the therapeutic polyphenols. The illustration features the specific sources: green tea leaves (EGCG), grapes (resveratrol), turmeric rhizomes (curcumin), apples (quercetin), celery (luteolin), and soybeans (genistein).

In recent years, considerable advancements have been achieved in the management of osteoarthritis by the utilization of polyphenols. A multitude of studies conducted both in vitro and in vivo, along with several clinical trials, have indicated that certain polyphenols may alleviate osteoarthritis symptoms. The major polyphenols and their dietary sources are highlighted in Figure 3.

### 3.1. EGCG

Epigallocatechin-3-gallate (EGCG), the principal bioactive catechin in green tea (Camellia sinensis), is notable for its potent antioxidant, anti-inflammatory, and chondroprotective properties [33,34,35].

In vitro studies with human primary chondrocytes and cartilage explants have given important mechanistic insights into the chondroprotective pathways of EGCG. Matrix degradation is mediated by the induction of matrix metalloproteinases (MMPs, particularly MMP-1, MMP-3, and MMP-13) and aggrecanases (ADAMTS-4 and ADAMTS-5) following activation by the pro-inflammatory cytokines interleukin-1 beta (IL-1β) and tumor necrosis factor-alpha (TNF-α) [33]. EGCG is known to effectively suppress the nuclear factor-kappa B (NF-κB) and mitogen-activated protein kinase (MAPK) signaling cascades, thereby attenuating IL-1β-induced expression of these catabolic enzymes [34]. EGCG also inhibits the induction of nitric oxide synthase (iNOS) and cyclooxygenase-2 (COX-2), leading to significant decreases in nitric oxide (NO) and prostaglandin E2 (PGE2) levels [35]. EGCG protects against chondrocyte apoptosis and maintains cellular homeostasis by reducing oxidative stress through ROS scavenging and activation of nuclear factor erythroid 2-related factor 2 (Nrf2) pathways [36].

The chondroprotective efficacy of EGCG shown in cellular models translates well to animal models of OA. The administration of EGCG consistently ameliorates joint degeneration in surgical rodent models, such as destabilization of the medial meniscus (DMM) and anterior cruciate ligament transection (ACLT), as well as in chemical intra-articular monoiodoacetate (MIA) models [37]. Oral or intra-articular administration of EGCG significantly reduces OARSI histology scores, prevents cartilage erosion, suppresses subchondral bone sclerosis, and reduces synovial inflammation [38]. Behavioural tests also demonstrate that EGCG alleviates tactile allodynia and joint pain, further confirming its analgesic properties in addition to its structural protection [39]. More recently, advanced delivery formulations such as microparticles [40,41] and nanoparticles [42] have further improved EGCG bioavailability and intra-articular residence time [40].

Preclinical evidence for EGCG is strong, but human data from clinical trials of green tea extract and standardized EGCG in patients with OA are required. Early clinical interventions have shown that knee OA patients receiving standardized green tea extracts rich in EGCG demonstrate significant improvements in WOMAC (Western Ontario and McMaster Universities Osteoarthritis Index) pain, stiffness, and physical function scores compared to placebo groups [41]. Biomarker analysis in clinical subjects shows reductions in systemic inflammatory markers, such as high-sensitivity C-reactive protein (hs-CRP), and in markers of cartilage degradation, such as serum COMP (cartilage oligomeric matrix protein) [42]. Despite these promising clinical signals, the systemic bioavailability remains a hurdle. Ongoing trials are investigating modified oral delivery systems and targeted intra-articular formulations to achieve optimal therapeutic concentration within the joint capsule [43].

To summarise, epigallocatechin-3-gallate (EGCG) is a promising therapeutic agent for the treatment of osteoarthritis. Extensive in vitro cellular studies, robust in vivo animal models, and emerging clinical trials support EGCG’s targeting of inflammatory, oxidative, and catabolic pathways to preserve joint integrity [42,43].

### 3.2. Resveratrol

Resveratrol (3,5,4′-trihydroxystilbene), a natural polyphenol phytoalexin, is abundant in grapes, red wine, berries, and peanuts and has been considered a promising candidate for OA management [44]. Resveratrol is well known for its pleiotropic biological properties, including anti-inflammatory, antioxidant, anti-apoptotic, and metabolic regulatory activities [45]. Resveratrol represents a promising therapeutic platform for the development of disease-modifying osteoarthritis drugs (DMOADs) by modulating key intracellular signaling hubs, such as silent information regulator 1 (SIRT1), nuclear factor-kappa B (NF-κB), AMP-activated protein kinase (AMPK), and nuclear factor erythroid 2-related factor 2 (Nrf2) [46].

Extensive cellular investigations using primary human chondrocytes and cartilage explants have delineated the multi-targeted chondroprotective mechanisms of resveratrol. In OA pathogenesis, pro-inflammatory cytokines such as interleukin-1 beta (IL-1β) and tumor necrosis factor-alpha (TNF-α) upregulate matrix metalloproteinases (MMPs, notably MMP-1, MMP-3, and MMP-13) and aggrecanases (ADAMTS-4 and ADAMTS-5), culminating in enzymatic digestion of type II collagen and proteoglycans within the extracellular matrix (ECM) [47].

Resveratrol directly counters cytokine-induced ECM degradation by activating SIRT1, an NAD+-dependent deacetylase [48]. Activated SIRT1 deacetylates the p65 subunit of NF-κB, thereby blocking NF-κB nuclear translocation and suppressing the downstream transcription of MMP-13, ADAMTS-5, inducible nitric oxide synthase (iNOS), and cyclooxygenase-2 (COX-2) [49]. Consequently, resveratrol significantly reduces inflammatory mediators, including nitric oxide (NO) and prostaglandin E2 (PGE2) [50]. Furthermore, resveratrol restores cellular redox homeostasis by mitigating reactive oxygen species (ROS) production and upregulating endogenous antioxidant enzymes (HO-1, SOD2) via AMPK/Nrf2 signaling pathways, ultimately preventing chondrocyte senescence and mitochondrial apoptosis [51].

The chondroprotective efficacy of resveratrol has been extensively validated in diverse animal models of OA, including surgical models such as destabilization of the medial meniscus (DMM) and anterior cruciate ligament transection (ACLT), as well as chemical models induced by intra-articular monoiodoacetate (MIA) [52]. Systemic oral administration or direct intra-articular injection of resveratrol consistently preserves the structural integrity of articular cartilage, reduces OARSI histological damage scores, and limits subchondral bone microstructural changes [53].

In rat and mouse DMM models, resveratrol treatment markedly suppresses cartilage matrix degradation and synovial lining hyperplasia through SIRT1-dependent suppression of synovial inflammation [54]. In MIA-induced post-traumatic OA models, resveratrol exhibits potent analgesic actions, suppressing mechanical hyperalgesia and weight-bearing asymmetry by attenuating peripheral neural sensitization and spinal microglial activation [55]. To overcome the rapid hepatic metabolism and poor aqueous solubility of resveratrol, advanced delivery vectors such as intra-articular sustained-release hydrogels, polymeric microparticles, and liposomal formulations have been developed, demonstrating prolonged joint retention, increased bioavailability, and better structural preservation than unformulated resveratrol [56].

Encouraging results have been obtained in translating preclinical findings to the human clinical setting. Early randomised, double-blind, placebo-controlled clinical trials have evaluated the therapeutic utility of resveratrol as an adjuvant therapy in patients with mild-to-moderate knee OA [57]. Standard treatment with meloxicam or acetaminophen with daily oral supplementation of resveratrol (500 mg/day for 90 days) significantly improved the reduction in Western Ontario and McMaster Universities Osteoarthritis Index (WOMAC) total scores, visual analog scale (VAS) pain scores, and joint stiffness compared to NSAID monotherapy in clinical trials [58].

The systemic and local anti-inflammatory mechanism of resveratrol is further supported by biomarker assessments in trial subjects. Resveratrol treatment led to a significant decrease in serum levels of pro-inflammatory cytokines (IL-1β, TNF-α, IL-6) and cartilage breakdown products, as well as a decrease in high-sensitivity C-reactive protein (hs-CRP) [59]. Moreover, safety evaluations showed good tolerability, with low rates of adverse events [60]. Nevertheless, future large-scale, long-term Phase III clinical trials using optimized, bioavailable formulations are necessary to establish standard clinical dosing regimens and to evaluate long-term structural changes in joint cartilage via magnetic resonance imaging (MRI) [61].

Resveratrol is a potent natural polyphenol with promise for disease-modifying effects in osteoarthritis. Resveratrol targets inflammatory, oxidative, and catabolic pathways for joint preservation with strong in vitro mechanisms, strong in vivo structural protection, and early clinical efficacy [62]. Further advancement of targeted intra-articular drug delivery systems will be crucial to achieving maximum therapeutic efficacy in clinical practice.

### 3.3. Curcumin

Curcumin is a major hydrophobic polyphenol obtained from the rhizome of turmeric (*Curcuma longa*) [63], and has been of considerable scientific interest in OA management. Curcumin is known for its multi-target biological activity and exhibits potent anti-inflammatory, antioxidant, anti-apoptotic, and chondroprotective effects [64]. Curcumin holds potential as a natural disease-modifying osteoarthritis drug (DMOAD) candidate through the regulation of key molecular targets such as nuclear factor-kappa B (NF-κB), cyclooxygenase-2 (COX-2), matrix metalloproteinases (MMPs), and reactive oxygen species (ROS) [65].

The cellular mechanisms of the chondroprotective effects of curcumin were investigated in primary human chondrocytes and cartilage explants. Pro-inflammatory cytokines, mainly interleukin-1 beta (IL-1β) and tumor necrosis factor-alpha (TNF-α), stimulate IκB kinase (IKK) and thus the nuclear translocation of NF-κB [66]. This cascade of transcriptional activation leads to the upregulation of catabolic enzymes, such as MMP-1, MMP-3, MMP-13, ADAMTS-4, and ADAMTS-5, that degrade type II collagen and aggrecan in the extracellular matrix (ECM) [67].

Curcumin inhibits IL-1β-induced phosphorylation of IκBα and nuclear translocation of NF-κB p65, leading to a significant reduction in MMP and ADAMTS gene expression [68]. In addition, curcumin suppresses the expression of inducible nitric oxide synthase (iNOS) and COX-2, thereby reducing nitric oxide (NO) and prostaglandin E2 (PGE2) levels [69]. Curcumin decreases intracellular ROS accumulation, protects mitochondrial function in chondrocytes, and prevents oxidative stress-induced apoptosis via the nuclear factor erythroid 2-related factor 2 (Nrf2) antioxidant axis [70].

The chondroprotective efficacy of curcumin has been demonstrated in several preclinical animal models of OA, including surgical models of destabilization of the medial meniscus (DMM), anterior cruciate ligament transection (ACLT), and chemical intra-articular monoiodoacetate (MIA) models [71]. Oral or intra-articular administration of curcumin maintains the structural integrity of the cartilage, reduces remodeling of the subchondral bone, and decreases synovial inflammation [72].

In rodent models, curcumin was shown to significantly reduce OARSI histological damage scores and systemic inflammatory markers [73]. Moreover, behavioral tests have shown that curcumin reduces tactile allodynia and joint pain [74]. To overcome the inherent limitations of curcumin, such as poor aqueous solubility, rapid metabolism, and low systemic bioavailability, researchers have developed advanced formulations, including phospholipid complexes (Meriva), nanoparticles, and bio-enhanced micellar systems, that provide enhanced intra-articular accumulation and superior structural protection [75].

The clinical utility of bioenhanced curcumin formulations in patients with knee OA is supported by translational evidence from randomized, double-blind, controlled clinical trials [76]. Clinical trials of standardized curcuminoids (Meriva, Theracurmin, C3 Complex, etc.) have shown significant reductions in pain and stiffness scores on the Western Ontario and McMaster Universities Osteoarthritis Index (WOMAC) and Visual Analog Scale (VAS) pain scores compared to placebo [77].

In head-to-head clinical comparisons, bio-enhanced curcumin formulations demonstrated clinical efficacy comparable to that of standard NSAIDs (e.g., ibuprofen and diclofenac) with significantly fewer gastrointestinal adverse events [78]. Patient assessments have consistently shown improved physical function and reduced need for rescue analgesics [79]. Biomarker assays in trial subjects confirm reductions in systemic inflammatory markers, including high-sensitivity C-reactive protein (hs-CRP) and serum COMP levels [80]. These findings support the use of bioavailable curcumin as an effective and well-tolerated option for long-term OA management [81].

Curcumin represents a viable natural polyphenol for osteoarthritis management. Supported by in vitro cellular pathway data, in vivo structural protection, and clinical trials of bio-enhanced formulations, curcumin targets inflammatory, catabolic, and oxidative cascades to maintain joint integrity and relieve pain [82]. Continued improvements in targeted intra-articular delivery will further strengthen its clinical application.

### 3.4. Quercetin

Quercetin (3,3′,4′,5,7-pentahydroxyflavone), a natural flavonoid polyphenol widely distributed in apples, onions, berries, tea, and red wine, has emerged as a promising candidate for OA therapy [83]. Renowned for its potent antioxidant, anti-inflammatory, anti-apoptotic, and novel senolytic properties, quercetin targets multiple altered molecular signaling cascades within the joint ecosystem [84]. By modulating nuclear factor-kappa B (NF-κB), nuclear factor erythroid 2-related factor 2 (Nrf2), sirtuin-1 (SIRT1), and the senescence-associated secretory phenotype (SASP), quercetin exhibits comprehensive chondroprotective potential [85].

In vitro experiments utilizing primary human chondrocytes, cartilage explants, and synovial cells have revealed the detailed molecular pathways through which quercetin preserves joint homeostasis. Pro-inflammatory cytokines, specifically interleukin-1 beta (IL-1β) and tumor necrosis factor-alpha (TNF-α), activate IκB kinase (IKK) and trigger the nuclear translocation of NF-κB p65 [86]. This cascade upregulates major matrix-degrading enzymes, including matrix metalloproteinases (MMP-1, MMP-3, and MMP-13) and aggrecanases (ADAMTS-4 and ADAMTS-5), leading to extracellular matrix (ECM) degradation [87].

Quercetin effectively inhibits IL-1β-induced NF-κB nuclear translocation, resulting in a marked downregulation of MMP-13 and ADAMTS-5 gene expression [88]. Furthermore, quercetin suppresses the expression of inducible nitric oxide synthase (iNOS) and cyclooxygenase-2 (COX-2), thereby significantly reducing the downstream production of nitric oxide (NO) and prostaglandin E2 (PGE2) [89]. Notably, quercetin acts as a potent activator of the Nrf2/HO-1 axis, enhancing endogenous antioxidant defenses, neutralizing reactive oxygen species (ROS), and protecting chondrocytes from oxidative stress-induced mitochondrial dysfunction and apoptosis [90]. In addition, quercetin functions as a senolytic agent by selectively eliminating senescent chondrocytes and suppressing SASP factors (e.g., IL-6, IL-8), thereby preventing the propagation of inflammation within the cartilage matrix [91].

The chondroprotective and disease-modifying efficacy of quercetin has been extensively demonstrated in preclinical animal models of OA, including surgical destabilization of the medial meniscus (DMM), anterior cruciate ligament transection (ACLT), and chemical intra-articular monoiodoacetate (MIA) models [92]. Systemic oral administration or direct intra-articular injection of quercetin significantly attenuates cartilage damage, suppresses subchondral bone remodeling, and decreases synovial inflammation [93].

In rat and mouse surgical OA models, quercetin treatment reduces OARSI histological scores, preserves proteoglycan content, and suppresses synovial lining hyperplasia by activating the SIRT1 and AMPK signaling pathways [94]. In MIA-induced post-traumatic OA models, quercetin has shown powerful analgesic effects, relieving mechanical allodynia and thermal hyperalgesia via inhibiting spinal neuroinflammation and peripheral pain sensitization [95]. Novel drug delivery technologies, such as quercetin-loaded polymeric nanoparticles, liposomes, and hyaluronic acid hydrogel carriers, have been developed to overcome quercetin’s poor aqueous solubility and rapid metabolic degradation [96]. These sophisticated systems prolong the intra-articular residence time, enhance the bioavailability, and provide localised long-term structural protection [97].

The translation of preclinical findings to clinical settings has yielded promising results. The efficacy of oral quercetin supplementation, alone or with bioavailability enhancers, such as phytosomes or rutin, has been evaluated in early randomized, double-blind, placebo-controlled clinical trials in patients with knee OA [98]. Clinical trials have shown that daily administration of quercetin (500–1000 mg/day) for 8 to 12 weeks significantly improves scores on the Western Ontario and McMaster Universities Osteoarthritis Index (WOMAC) for pain, stiffness and physical function compared with placebo [99].

Biomarker assessments in clinical trial participants validate the systemic anti-inflammatory effects of quercetin by demonstrating significant decreases in serum high-sensitivity C-reactive protein (hs-CRP), TNF-α, and cartilage degradation markers such as serum COMP [100]. The combination of quercetin with senolytic regimens (e.g., Dasatinib plus Quercetin, D+Q) is being intensely investigated in clinical trials for age-related joint degeneration, where remarkable reductions in senescent cell burden and improvements in joint mobility have been observed [101]. Clinical safety monitoring confirms its high tolerability and negligible adverse effects, supporting the use of quercetin as a safe adjuvant in the long-term management of OA [102].

Quercetin is a versatile natural polyphenol with considerable therapeutic promise in the management of osteoarthritis. Quercetin is beneficial for targeting oxidative stress, catabolic degradation, inflammation, and cellular senescence with strong in vitro cellular signaling mechanisms, compelling in vivo structural and analgesic benefits, and increasing evidence from clinical trials [103]. Continued development of bioenhanced delivery formulations will further enable clinical translation of this work as a DMOAD.

### 3.5. Luteolin

Luteolin (3′,4′,5,7-tetrahydroxyflavone) is a natural flavone abundant in celery, green peppers, broccoli, parsley, thyme and chamomile [104]. Luteolin is well known for its multifaceted biological activities, including anti-inflammatory, antioxidant, anti-apoptotic, and chondroprotective effects, and it acts on key signaling cascades involved in the pathogenesis of OA [105]. Luteolin modulates nuclear factor kappa B (NF-κB), mitogen-activated protein kinase (MAPK), sirtuin-1 (SIRT1), and Nrf2 pathways to protect chondrocytes and attenuate synovial inflammation [106].

The cellular mechanisms underlying the chondroprotective effects of luteolin have been delineated in vitro using human and rodent chondrocytes, synovial fibroblasts, and joint explants. In OA, pro-inflammatory cytokines, such as interleukin-1 beta (IL-1β) and tumor necrosis factor-alpha (TNF-α), activate the IκB phosphorylation and nuclear translocation of NF-κB p65 and p38 MAPK, ERK, and JNK pathways [107]. This signaling cascade upregulates major matrix metalloproteinases (MMP-1, MMP-3, MMP-13) and aggrecanases (ADAMTS-4, ADAMTS-5), leading to the breakdown of collagen type II and proteoglycans [108].

Luteolin treatment effectively inhibited IL-1β- and TNF-α-induced activation of NF-κB and MAPK signaling pathways in primary human chondrocytes [109]. This inhibition substantially suppresses the transcriptional activation of MMP-13 and ADAMTS-5, thereby inhibiting extracellular matrix degradation [110]. Furthermore, luteolin downregulates the expression of inducible nitric oxide synthase (iNOS) and cyclooxygenase-2 (COX-2), thereby reducing the accumulation of nitric oxide (NO) and prostaglandin E2 (PGE2) [111]. Luteolin also activates the Nrf2/HO-1 antioxidant defense signaling network, which reduces ROS levels and maintains mitochondrial membrane potential, thus preventing ROS-mediated chondrocyte apoptosis [112]. Additionally, luteolin inhibits cellular senescence markers and inflammatory senescence-associated secretory phenotype (SASP) factors (e.g., IL-6, IL-8), maintaining chondrocyte phenotype and cellular survival [113].

Luteolin’s disease-modifying potential has been validated in various animal models of OA, including surgical models such as destabilization of the medial meniscus (DMM) and anterior cruciate ligament transection (ACLT), as well as chemical models such as intra-articular monoiodoacetate (MIA) injection [114]. Oral or localised intra-articular administration of luteolin consistently inhibits cartilage degeneration, subchondral bone sclerosis, and synovial hypervascularization [115].

Luteolin therapy downregulates the NLRP3 inflammasome and upregulates SIRT1 expression, resulting in reduced Osteoarthritis Research Society International (OARSI) histological scores, preservation of cartilage thickness, and restoration of proteoglycan staining intensity in DMM and ACLT rodent models [116]. Luteolin showed anti-nociceptive efficacy in MIA-induced post-traumatic OA models by significantly reversing weight-bearing asymmetry, mechanical allodynia and thermal hyperalgesia through attenuation of peripheral joint inflammation and DRG neuroinflammation [117]. To overcome luteolin’s low water solubility and poor systemic bioavailability, advanced delivery formulations have been developed, including luteolin-loaded polymeric nanoparticles, liposomes and hyaluronic acid hydrogel matrices [118]. These novel intra-articular drug delivery mechanisms increase retention time within the joint space, thereby improving long-term therapeutic response and preserving cartilage structure [119].

The clinical assessment of luteolin, primarily as an active component of standardized botanical extracts or bio-enhanced flavonoid complexes, provides promising translational evidence for the treatment of OA [120]. Randomised double-blind placebo-controlled trials of luteolin-rich natural extracts (alone or synergistically combined with bio-enhancers such as lecithin phytosomes or rutin) in patients with knee OA have shown significant clinical benefits [121]. Patients receiving luteolin intervention daily (150–500 mg/day for 8–12 weeks) showed statistically significant reductions in Western Ontario and McMaster Universities Osteoarthritis Index (WOMAC) scores for pain, joint stiffness, and physical disability [122].

Systemic biomarker studies in clinical trial groups demonstrated significant reductions in circulating inflammatory cytokines (TNF-α, IL-6), serum high-sensitivity C-reactive protein (hs-CRP), and markers of cartilage turnover including serum COMP and CTX-II [123]. Safety assessments in clinical studies have confirmed the excellent tolerability of luteolin, with no serious adverse reactions reported [124], thereby supporting its use as a safe, effective nutraceutical adjunct for OA treatment.

Luteolin is a promising natural polyphenol candidate for osteoarthritis treatment. Luteolin is an effective agent against matrix degradation, oxidative stress, cellular senescence, and inflammation, supported by strong in vitro mechanistic evidence, robust in vivo structural protection, and positive clinical trial results [125]. Continued progress in bio-targeted nano-formulations will further optimize its translation as a clinical DMOAD.

### 3.6. Genistein

Genistein (4′,5,7-trihydroxyisoflavone), a naturally occurring phytoestrogenic flavonoid found predominantly in soybeans (Glycine max), has emerged as a promising candidate [126]. Genistein has great potential to mitigate cartilage degradation and joint deterioration due to its potent anti-inflammatory, antioxidant, anti-apoptotic, and phytoestrogenic activities [127].

Studies in vitro with primary human or rodent chondrocytes and synovial fibroblasts have been crucial in unraveling the complex biochemical pathways targeted by genistein. Classic cell-based models that mimic OA inflammatory microenvironments are primary chondrocytes stimulated with IL-1β or TNF-α [128]. IL-1β exposure triggers a strong inflammatory cascade, leading to the upregulation of MMP-1, MMP-3, MMP-13, ADAMTS-4, and ADAMTS-5, which together degrade type II collagen and aggrecan, the major structural components of the ECM [129].

Genistein treatment effectively suppresses IL-1β-induced expression of these matrix-degrading enzymes in a dose-dependent manner [130]. Molecular analyses demonstrate that genistein inhibits the nuclear factor kappa B (NF-κB) signaling pathway by preventing the phosphorylation and degradation of inhibitor of kappa B alpha (IκBα) and suppressing the nuclear translocation of the p65 subunit [131]. Concurrently, genistein attenuates the activation of mitogen-activated protein kinase (MAPK) signaling cascades, specifically suppressing the phosphorylation of extracellular signal-regulated kinase (ERK), c-Jun N-terminal kinase (JNK), and p38 MAPK [132].

In addition to inhibiting catabolic pathways, genistein exerts profound antioxidant and anti-apoptotic actions. In chondrocytes exposed to hydrogen peroxide or inflammatory cytokines, genistein reduces intracellular reactive oxygen species (ROS) levels and restores mitochondrial membrane potential [133]. It suppresses the expression of inducible nitric oxide synthase (iNOS) and cyclooxygenase-2 (COX-2), leading to a significant reduction in nitric oxide (NO) and prostaglandin E2 (PGE2) production [134]. Furthermore, genistein modulates the Bcl-2 family proteins—upregulating the anti-apoptotic Bcl-2 and downregulating the pro-apoptotic Bax—and inhibits the activation of caspase-3 and caspase-9, thereby preserving chondrocyte viability [135]. As an estrogen receptor agonist (specifically showing higher selectivity for ER-β), genistein activates ER-mediated signaling, promoting chondrocyte proliferation and ECM synthesis [136].

The protective effect of genistein exhibited in vitro has been convincingly translated to animal models of OA. Preclinical studies are mainly based on surgical models such as destabilization of the medial meniscus (DMM) or anterior cruciate ligament transection (ACLT), and chemical models induced by intra-articular injection of monosodium iodoacetate (MIA) [137].

Oral or systemic genistein administration significantly reduced cartilage damage, as evidenced by lower Osteoarthritis Research Society International (OARSI) histological scores and Mankin scores in rats and mice with surgically induced OA [138]. Histopathological assessments indicate that genistein-treated animals maintain cartilage structural integrity, exhibit increased proteoglycan content, and show reduced loss of superficial and middle chondrocytes [139]. Immunohistochemical analyses demonstrate significant downregulation of MMP-13 and upregulation of collagen type II and aggrecan in articular cartilage tissues [135].

Genistein showed significant anti-inflammatory effects in the MIA-induced rat OA model that mimics inflammatory degradation [140]. Orally administered genistein reduces synovial hypercellularity, suppresses joint swelling, and reduces the concentrations of pro-inflammatory cytokines (IL-1β, TNF-α, IL-6) in synovial fluid [141]. Genistein has been reported to possess anti-resorptive and bone-sparing effects in animal models [142]. Moreover, genistein has a protective effect on the remodelling of subchondral bone by preventing subchondral bone loss and microarchitectural deterioration through modulation of the RANKL/OPG ratio [143].

Soy isoflavones, particularly genistein, have been examined in a number of human trials and epidemiological studies in patients with OA or in post-menopausal women with joint discomfort, so as to translate the pre-clinical promise into clinical efficacy [144]. Postmenopausal women are especially vulnerable to rapid OA progression due to oestrogen deficiency, which highlights the need for phytoestrogenic intervention [145].

Clinical trials of genistein-rich soy isoflavone supplementation in patients with knee OA have shown improvements in pain scores, joint stiffness, and physical function, as assessed by the Western Ontario and McMaster Universities Osteoarthritis Index (WOMAC) and the Visual Analog Scale (VAS) [146]. Randomized, double-blind, placebo-controlled trials have demonstrated that daily oral administration of soy extract containing genistein for 12–24 weeks resulted in statistically significant reductions in serum markers of inflammation (high-sensitivity C-reactive protein, hs-CRP) and markers of cartilage turnover (C-telopeptide of type II collagen, CTX-II) as compared to placebo [147].

Clinical trials have also shown that genistein is well tolerated with a good safety profile and causes fewer gastrointestinal side effects than traditional NSAIDs [148]. However, researchers note variability in clinical response among individuals, which may be due to differences in the composition of the gut microbiota required to metabolize genistein into its active metabolites, as well as limitations in the oral bioavailability of raw genistein [149].

In summary, the natural polyphenol genistein exhibits robust, multi-faceted protective efficacy against osteoarthritis across in vitro, in vivo, and clinical domains [150]. By concurrently modulating inflammatory signaling cascades (NF-κB, MAPK), inhibiting catabolic matrix-degrading enzymes (MMPs, ADAMTS), reducing oxidative stress and chondrocyte apoptosis, and preserving subchondral bone integrity, genistein addresses multiple core aspects of OA pathology [151]. Clinical studies further substantiate its capacity to reduce joint pain and functional impairment, with a high safety profile. Future research should focus on novel drug delivery systems, such as nano-formulations or intra-articular sustained-release hydrogels, to overcome bio-accessibility constraints and optimize its therapeutic translation as a true disease-modifying osteoarthritis drug [152].

## 4. Clinical Evidence of Natural Polyphenols Against Osteoarthritis

Over the past decade, natural polyphenols have been extensively evaluated in clinical settings for their therapeutic potential as disease-modifying or symptom-modifying nutraceuticals in knee osteoarthritis (OA). Below (Table 1) is a structured summary of clinical trials conducted during the last decade evaluating epigallocatechin gallate (EGCG), resveratrol, curcumin, quercetin, luteolin, and genistein.

### 4.1. Polyphenols (EGCG, Resveratrol, Curcumin, Quercetin, Luteolin, and Genistein) Combined with Conventional Pharmaceuticals in the Management of Osteoarthritis

Osteoarthritis (OA) is a prevalent, debilitating degenerative joint disorder characterized by progressive cartilage degradation, subchondral bone remodeling, and persistent synovial inflammation [6]. Standard pharmacotherapeutic interventions are targeted mainly toward symptomatic pain relief rather than reversal of structural tissue damage and include predominantly nonsteroidal anti-inflammatory drugs (NSAIDs), intra-articular corticosteroids, and analgesics [165]. Moreover, the long-term clinical use of conventional NSAIDs is frequently limited by serious gastrointestinal, renal and cardiovascular toxicities [166]. To overcome these therapeutic limitations, translational research has recently increasingly focused on the co-administration of natural polyphenols, such as epigallocatechin gallate (EGCG), resveratrol, curcumin, quercetin, luteolin, and genistein, with standard OA drugs [167]. These pleiotropic phytochemicals have strong anti-inflammatory, antioxidant and chondroprotective effects that complement the standard drug regimens [168]. In vitro molecular studies have shown that the combination of polyphenols with NSAIDs (e.g., celecoxib, meloxicam or diclofenac) or symptomatic slow-acting drugs for OA (SYSADOAs such as glucosamine and chondroitin sulfate) exerts a potent multi-target synergy [169]. In particular, EGCG, resveratrol, and curcumin inhibit nuclear factor kappa B (NF-κB) p65 phosphorylation, thereby reducing IL-1β-induced expression of catabolic enzymes (matrix metalloproteinases (MMP-1, MMP-3, MMP-13) and ADAMTS-5) in human chondrocytes [170]. Concurrently, quercetin, luteolin, and genistein scavenge reactive oxygen species (ROS), inhibit cyclooxygenase-2 (COX-2) and inducible nitric oxide synthase (iNOS), and preserve extracellular matrix integrity by upregulating aggrecan and type II collagen synthesis [171].

In addition, preclinical in vivo studies in animal models of OA, including surgical induction of destabilization of the medial meniscus (DMM) or injection of monosodium iodoacetate (MIA), also support the disease-modifying and organ-protective effects of polyphenol-pharmaceutical combinations [172]. Co-administration of oral curcumin with subtherapeutic doses of NSAID in MIA-induced rodent models produced comparable or greater attenuation of pain and functional mobility than full-dose NSAID monotherapy [173]. Histological assessments consistently show lower OARSI and Mankin scores in combination-treated cohorts, indicating preserved articular cartilage thickness, reduced osteophyte formation, and reduced proteoglycan loss [174]. Furthermore, intra-articular delivery platforms like hyaluronic acid hydrogels functionalized with EGCG or quercetin prolong intra-articular retention, inhibit synovial membrane hypervascularization, and reduce subchondral bone osteoclastogenesis by downregulating the RANKL/OPG ratio [175]. Importantly, the concomitant administration of genistein mitigates adverse drug effects; enhance gastric mucosal defenses and antioxidant capacity and significantly reduce the incidence and severity of NSAID-induced gastric ulceration and hepatic oxidative stress in vivo [176].

Translational human clinical trials support that adding natural polyphenols to standard clinical management regimens significantly improves patient outcomes in primary knee OA [177]. Furthermore, double-blind, randomized, placebo-controlled clinical trials have shown that the addition of oral curcumin or resveratrol to conventional NSAID regimens for 6 to 12 weeks results in significantly greater reductions in Visual Analogue Scale (VAS) pain intensity and Western Ontario and McMaster Universities Osteoarthritis Index (WOMAC) subscores for stiffness and physical function than NSAID use alone [178]. Importantly, co-therapy was associated with a reduction in the daily NSAID dose by up to 50% without compromising clinical analgesic efficacy and with a significant decrease in the incidence of gastrointestinal complications [179]. Serum biomarker profiling of trial participants revealed pronounced post-treatment decreases in systemic inflammatory markers, including high-sensitivity C-reactive protein (hs-CRP), TNF-α, and IL-6, as well as reductions in cartilage breakdown markers such as C-terminal telopeptide of type I collagen [180]. Additionally, clinical evaluations of quercetin and EGCG formulations administered as adjuvants to intra-articular hyaluronic acid injections or to oral glucosamine demonstrated improvements in joint biomechanics and patient-reported quality of life [181]. Collectively, evidence across in vitro, in vivo, and clinical domains underscores that combining EGCG, resveratrol, curcumin, quercetin, luteolin, and genistein with conventional medicines represents a highly viable, synergistic strategy for multi-targeted OA management [182].

Apart from conventional NSAIDs, fixed-dose combination analgesics such as tra-madol/paracetamol (75 mg/650 mg) are often used for the management of early stage symptomatic knee OA [183]. Recent real-world studies provide important pragmatic insights into these regimens. Conforti et al. [183] conducted a retrospective observational cohort study that demonstrated significant and rapid pain relief (VAS score) and functional improvement (WOMAC index) in patients with early symptomatic knee OA receiving a short-term, 10-day treatment with fixed-dose tramadol/paracetamol [183]. On the basis of these results, a single-center retrospective extension study by Cipolloni et al. [184] confirmed the maintenance of these symptomatic and functional improvements at short-term (1 month) and mid-term (3 months) follow-up without major safety events [184]. Nevertheless, these results remain exploratory, because the studies were retrospective and uncontrolled observations. They highlight the clinical utility of short-term multi-target control of pain, but also the need for long-term multi-modal strategies (e.g., polyphenol supplementation) to achieve sustained management without long-term opioid-related or analgesic tolerance risks.

Table 2 outlines preclinical and clinical studies evaluating natural polyphenol-pharmaceutical combination therapies in osteoarthritis management.

### 4.2. Polyphenol-Based Interventions vs. Current Real-World Pharmacological Management of Osteoarthritis

In daily clinical practice, the choice of which drug to use in patients with OA is not only based on the initial analgesic efficacy, but also on the multimorbidity of the patients, the renal function, the gastrointestinal (GI) and cardiovascular (CV) safety profiles, polypharmacy and the risks associated with long-term analgesic exposure [8,166]. Nonsteroidal anti-inflammatory drugs (NSAIDs) still remain a mainstay for acute flares and short-term pain relief [6,7], but chronic use is often limited by well-known toxicities such as GI mucosal ulceration, acute kidney injury, hypertension, and negative cardiovascular events [8,10,166]. A full network meta-analysis of NSAIDs, opioids and paracetamol for knee and hip OA found that while best performing NSAIDs are effective for short term pain relief, they have considerable safety trade-offs with long term use [10]. Weak opioids (e.g., tramadol) and combination opioid/analgesic regimens are short- to mid-term options for pain management [2,3], but their real-world utility is often limited by poor long-term treatment persistence, side effects of the central nervous system (nausea, dizziness, somnolence), physical dependence and increasing analgesic tolerance [2,3,10].

In this respect, bioavailable natural polyphenols (such as bio-enhanced curcumin, resveratrol, epigallocatechin-3-gallate (EGCG), quercetin, luteolin, and genistein) have a unique therapeutic profile [7,168]. Polyphenols not only provide short-term symptomatic analgesia but also modulate the underlying inflammatory, oxidative, and catabolic cascades driving OA progression, while displaying favourable systemic safety and high treatment persistence [7,9,199,200]. Moreover, clinical trials draw attention to their use as effective “analgesic-sparing” or adjunctive therapies that allow dose reductions in conventional NSAIDs of up to 50% without loss of pain control, thus reducing long-term pharmaceutical toxicity [178,179]. Based on the discussion above, Table 3 summarizes an overview of the clinical and pharmacologic characteristics of conventional medications for OA compared with polyphenol-based interventions.

## 5. Efficacy and Safety of Natural Polyphenols in the Treatment of Osteoarthritis

The fundamental toxicity models and safety testing criteria for anti-inflammatory compounds originated in early pharmacological studies dating back to the mid-1950s [199]. To circumvent these systemic toxicities, natural polyphenols—such as epigallocatechin-3-gallate (EGCG), resveratrol, curcumin, quercetin, luteolin, and genistein—have attracted substantial research attention as novel, multi-target disease-modifying arthritis drugs [200].

### 5.1. In Vitro Cytotoxicity and Biphasic Dose–Response (Hormesis)

A thorough understanding of polyphenol safety requires rigorous evaluation of their cytotoxicity profiles in articular joint tissues. In vitro investigations using primary human articular chondrocytes, synovial fibroblasts, and SW1353 cell lines consistently demonstrate a distinct hormetic (biphasic) dose–response relationship. At physiological or low micromolar concentrations, polyphenols exhibit robust cytoprotective and anti-catabolic properties [201]. They effectively downregulate interleukin-1 beta (IL-1β)-induced matrix metalloproteinases (MMP-1, MMP-3, MMP-13) and aggrecanases (ADAMTS-4, ADAMTS-5) by suppressing nuclear factor kappa B (NF-κB) and mitogen-activated protein kinase (MAPK) signaling cascades [201,202]. Conversely, exposure to supra-therapeutic or high micromolar concentrations (>50–100 µM) can induce pro-oxidant cellular stress. High intracellular polyphenol levels promote excessive accumulation of reactive oxygen species (ROS), disruption of mitochondrial membrane potential, cytochrome c release, and subsequent caspase-3/9-mediated apoptosis [203,204]. Thus, maintaining local drug concentrations strictly within the therapeutic window is vital to avoid cytotoxic responses.

### 5.2. In Vivo Safety Profile and Advanced Bio-Delivery Systems

Preclinical in vivo studies in rodent OA models, such as anterior cruciate ligament transection (ACLT), destabilization of the medial meniscus (DMM), and monosodium iodoacetate (MIA) induction, have demonstrated that oral administration of natural polyphenols has a favorable safety profile and low hepatic and renal toxicity [205]. Furthermore, the association of polyphenols with lower doses of classical NSAIDs effectively reduces gastric mucosal erosion and cardiovascular risk [206]. However, translation into human clinical practice is often limited by poor aqueous solubility, rapid phase II metabolic conjugation, low oral bioavailability, and short intra-articular half-life. Current pharmaceutical approaches to overcome these pharmacokinetic limitations without increasing cytotoxicity rely on advanced nanocarriers, liposomal formulations, polymeric nanoparticles, and biofunctionalized hyaluronic acid hydrogels [207]. Intra-articular administration of nano-formulations loaded with polyphenols enables sustained release and prolonged retention within the joint cavity, providing localized therapeutic efficacy while minimizing off-target systemic toxicity and optimizing the overall safety of treatment [208].

### 5.3. Bioavailability and Pharmacokinetics of Natural Polyphenols in Osteoarthritis

**Figure 4:**
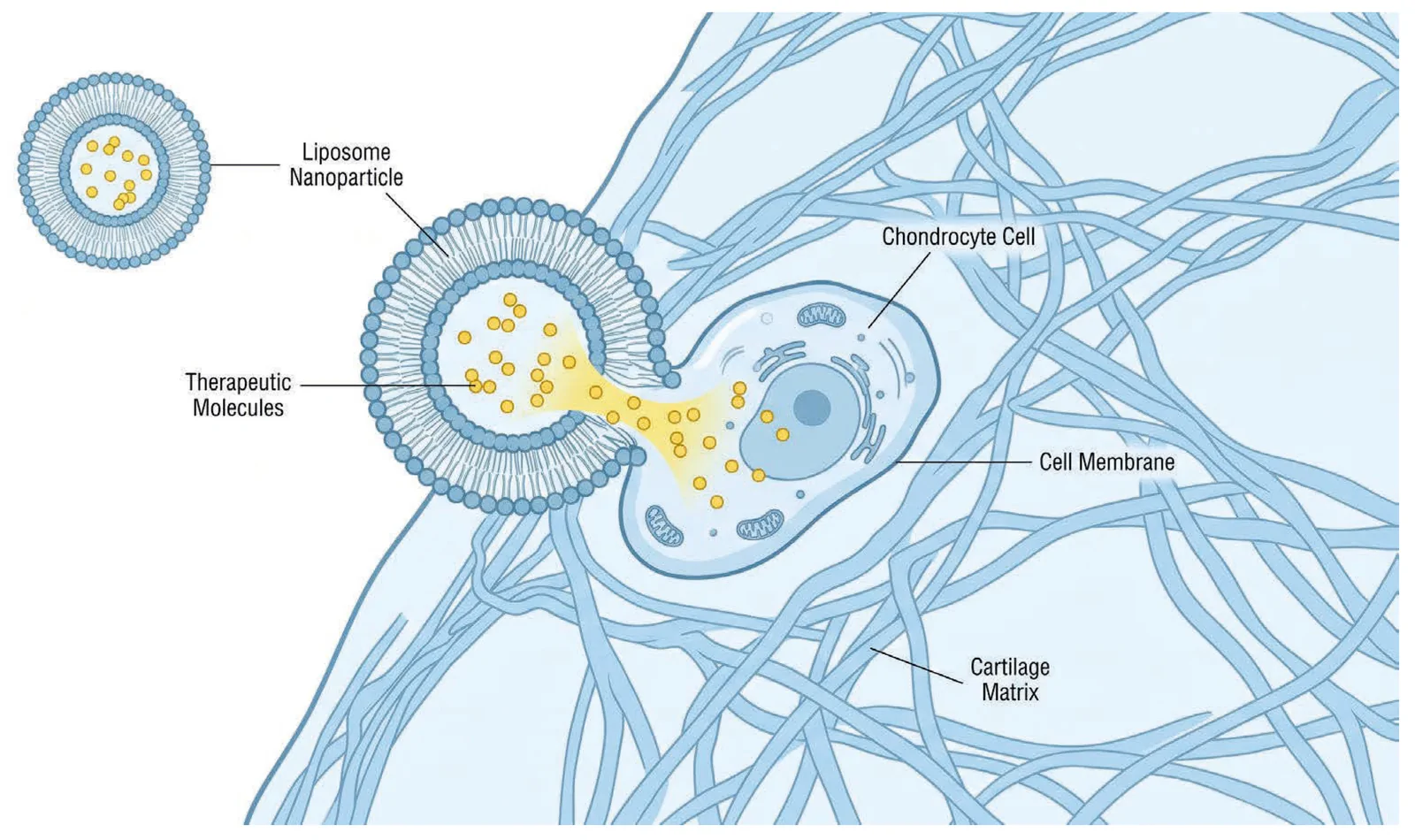
Liposomal Nanoparticles in Targeted Drug Delivery. Illustration showing how advanced nano-delivery platforms, such as liposomes, can penetrate the cartilage extracellular matrix and deliver bioavailable polyphenol molecules to target chondrocytes.

Natural polyphenols—such as curcumin, resveratrol, epigallocatechin-3-gallate (EGCG), quercetin, luteolin, and genistein—have demonstrated compelling chondroprotective, anti-inflammatory, and antioxidant activities in vitro. However, their clinical application as disease-modifying osteoarthritis drugs (DMOADs) is severely hampered by unfavorable pharmacokinetic profiles. The main biopharmaceutical problems are poor oral absorption, poor aqueous solubility, instability at physiological pH, rapid intestinal degradation, and extensive phase II metabolic transformation (glucuronidation and sulfation) in the liver and gut epithelium [209]. Thus, oral administration yields very low systemic concentrations; therefore, the active molecule does not effectively penetrate the avascular articular cartilage matrix [210]. Although native polyphenols reach a rapid maximum plasma concentration (Cmax) after oral ingestion, circulating levels are generally in the low nanomolar range, far below the micromolar levels required to inhibit inflammatory cascades such as NF-κB and MAPK [211]. Furthermore, free polyphenols are rapidly bound to plasma proteins, hence decreasing the free concentration in the synovial fluid. Another challenge is the direct intra-articular clearance pathways, as unformulated small-molecule polyphenols injected directly into the joint capsule are subject to rapid lymphatic drainage and elimination via blood capillaries, leading to a short half-life (t_1/2_) within the synovial space [212]. To sustain therapeutic efficacy, local bio-retention should be extended to maintain therapeutic concentrations and minimize systemic clearance [213]. To tackle these biopharmaceutical challenges, innovative pharmaceutical formulations have been devised (Figure 4). Phytosomes, liposomes, polymeric nanoparticles, and self-emulsifying pharmaceutical delivery systems (SEDDS) improve intestinal mucosal transport and protect polyphenols from enzymatic degradation [214]. At the same time, intra-articular injectable hydrogels, such as biofunctionalized hyaluronic acid matrices, can act as controlled-release depots, extending drug residence time in the joint space from hours to several weeks [215]. These bioengineered delivery systems greatly increase intra-articular bioavailability, allowing stable micromolar drug concentrations at the target chondrocytes, reducing the frequency of dosing and maximizing long-term therapeutic outcomes in OA management [216].

## 6. Limitations in Design of Clinical Studies and Direct Application of Natural Polyphenols in Osteoarthritis

Although natural polyphenols like curcumin, resveratrol, epigallocatechin-3-gallate (EGCG), and quercetin exhibit promising chondroprotective and anti-inflammatory properties in preclinical models, translating these findings into clinical practice faces substantial hurdles. A primary limitation is the pronounced heterogeneity in study design across clinical trials [217]. Existing human studies are frequently constrained by small cohort sizes, brief follow-up periods, and a lack of standardized dosing regimens. In addition, patient selection rarely considers OA phenotypes (e.g., inflammatory, mechanical, or metabolic subtypes), resulting in substantial interindividual variability in treatment response [218]. Many trials rely on subjective patient-reported outcome measures, such as WOMAC or VAS pain scores, and do not include objective imaging biomarkers or biochemical assays to monitor structural changes in the joint [219]. The direct therapeutic application of unformulated natural polyphenols [220] is limited by poor oral bioavailability and rapid Phase II metabolism. In the liver, native polyphenols are extensively glucuronidated and sulfated, resulting in low levels of active compounds in circulating plasma and in the avascular articular cartilage matrix [221]. Therefore, supra-physiological oral doses are necessary to attain therapeutic tissue levels, which increases the risk of gastrointestinal discomfort and off-target cytotoxicity [222]. Such translational bottlenecks are not resolved by direct intra-articular injections of unformulated polyphenols, since free small molecules are rapidly eliminated via the synovial microvasculature and lymphatic drainage, thereby drastically reducing their intra-articular residence time [218]. These translational obstacles are further compounded by the wide range of variability in polyphenol source materials, extraction protocols, and botanical standardization [223]. Commercial preparations often lack rigorous quantification of active bio-constituents, complicating cross-study comparisons and reproducible dosing. Future clinical trials should employ standardized high bioavailability delivery vehicles such as liposomes, phytosomes, and hydrogels to establish natural polyphenols as viable disease-modifying osteoarthritis drugs (DMOADs) with validated imaging and soluble biomarkers and standardized phenotype stratification [224,225].

## 7. Conclusions and Future Perspective

When evaluating polyphenol-based interventions in combination with conventional OA pharmacotherapy, it is important to distinguish between the pharmacological “synergy” and “additive or adjunctive” therapeutic effects. In preclinical cell and tissue models, true biochemical synergy is often observed, with polyphenols directly interacting with overlapping signalling hubs (e.g., EGCG or curcumin suppressing NF-κB and MAPK pathways in addition to COX-2 inhibition by NSAIDs). Clinical evidence, however, mostly suggests an additive or adjunctive (NSAID-sparing) effect. Thus, the co-administration of bioavailable polyphenols in human trials allows significant reductions in conventional NSAID dosages without compromising clinical analgesia, thereby minimising dose-dependent gastrointestinal and renal toxicities.

Moreover, it is important to clarify the levels of evidence of the current literature: although preclinical in vitro and in vivo animal models provide strong proof-of-concept for DMOAD actions (e.g., cartilage structural preservation, MMP-13/ADAMTS-5 inhibition, and anti-senescent effects), current clinical evidence in humans mainly considers polyphenols as symptomatic adjuncts and NSAID-sparing treatments. To prove pol-y-phenols as genuine clinical DMOADs, long-term, strict Phase III randomized controlled trials with objective structural endpoints, such as quantitative MRI and validated fluid biomarkers are required.

Natural polyphenols—including epigallocatechin-3-gallate (EGCG), resveratrol, curcumin, quercetin, luteolin, and genistein—demonstrate significant potential as multi-targeted disease-modifying osteoarthritis drugs (DMOADs) by suppressing nuclear factor-kappa B (NF-κB) and mitogen-activated protein kinase (MAPK) signaling pathways, reducing reactive oxygen species (ROS) accumulation, downregulating matrix-degrading enzymes (MMP-13, ADAMTS-5), and mitigating chondrocyte senescence and synovial inflammation. Furthermore, their use as synergistic adjuvants with reduced doses of conventional non-steroidal anti-inflammatory drugs (NSAIDs) or symptomatic slow-acting drugs for osteoarthritis (SYSADOAs) consistently relieves joint pain and improves physical function while decreasing gastrointestinal and systemic toxicities. The translation of these preclinical findings into definitive clinical practice in the future will require overcoming major pharmacokinetic limitations, including low oral bioavailability, rapid Phase II metabolism, short intra-articular residence times, and potential pro-oxidant cytotoxicity at supra-therapeutic concentrations. Future research should focus on long-term, large-scale Phase III clinical trials using standardized bio-enhanced delivery platforms, such as liposomes, polymeric nanoparticles, phytosomes, and intra-articular sustained-release hydrogels, to ensure prolonged local bioretention and targeted therapeutic efficacy. Moreover, to confirm structural cartilage preservation and establish these botanical agents as safe, standard treatment options for OA management, tailoring polyphenol-based regimens to specific patient endotypes and combining quantitative imaging with soluble molecular biomarkers will be critical.

## Figures and Tables

**Table 1:** Clinical Studies Evaluating the Efficacy and Molecular Impacts of Polyphenol Therapy in Patients with Osteoarthritis.

Polyphenol	Study Design	Dosage and Duration	Clinical Outcomes	Key Biomarker Changes	Reference
Epigallocatechin Gallate (EGCG)	Randomized controlled trial/In vitro cellular study using normal bovine and osteoarthritic human chondrocytes (cartilage cells)	100 mg to 800 mg/day for varying durations (grouped by ≤8 weeks or >8 weeks)/1 µg/mL to 4 µg/mL incubated for 24 to 48 h	Showed targeted anti-inflammatory benefits for metabolic syndrome/demonstrated a synergistic protective effect on cartilage cells against inflammation	Significantly decreased TNF-α (no significant overall effect on CRP or IL-6)/significantly reduced expression and production of IL-6, NO (Nitric Oxide), PGE2, MMP-3, and COX-2	[153,154]
Resveratrol	Pilot clinical study (patients with mild-to-moderate knee osteoarthritis)	500 mg/day (single oral dose) for 90 days	Promising therapeutic efficacy as part of multi-component therapeutic strategies	Associated with the reduction of inflammatory mediators and oxidative stress factors, while regulating metabolic signaling cascades (such as PI3K/AKT/mTOR and HIF-1/VEGF)	[155,156]
Curcumin	Multicenter, randomized, double-blind, placebo-controlled trial	250 mg once daily (providing 150 mg of curcuminoids) for 90 days/recommend supplementation for more than 12 weeks for optimal results	Significantly alleviated chronic knee pain and improved physical joint function/Significantly reduced joint pain, stiffness, and physical dysfunction. The extract demonstrated similar efficacy to NSAIDs in symptom relief but with a notably lower incidence of adverse side effects	Showed improvement (reduction) in systemic inflammatory markers (CRP, ESR) and cartilage degradation markers (GAGs) compared to the placebo group/significant reductions in VAS and WOMAC scores	[157,158]
Quercetin	Randomized, double-blind, placebo-controlled clinical trial in patients with symptomatic knee osteoarthritis/In vivo preclinical animal study (mouse model of carrageenan-induced inflammatory pain)	45 mg/day of quercetin glycosides (combined with 1200 mg glucosamine and 60 mg chondroitin) for 16 weeks/Acute doses administered orally and intraperitoneally (typically single doses evaluated over hours)	Significantly decreased the intensity of knee OA-associated clinical symptoms and improved physical function compared to the placebo/exerted potent antinociceptive (analgesic) effects, significantly reducing hyperalgesia and inflammatory pain	Demonstrated a trend of improvement in the balance of type II collagen synthesis and degradation (cartilage metabolism)/Inhibited the production of the pro-inflammatory cytokine IL-1β and reduced oxidative stress by preventing the depletion of reduced glutathione (GSH)	[159,160]
Luteolin	Network meta-analysis of Randomized Controlled Trials (RCTs) evaluating botanical extracts for knee OA	Meta-analysis of various botanical formulations; specific isolated luteolin dosages were not the primary focus	Demonstrated significant potential in managing knee osteoarthritis, primarily by improving joint pain and physical function with a favorable safety profile and minimal adverse effects	Anti-inflammatory effects by inhibiting major inflammatory signaling pathways (such as NF-κB and MAPK) and subsequently decreasing the production of pro-inflammatory cytokines (IL-1β, TNF-α, IL-6, and IL-8) and inflammatory enzymes such as COX-2. It also reduces oxidative stress markers (ROS)	[161,162]
Genistein	Clinical trial evaluation on menopausal symptoms/review of emerging natural-product-based treatments for osteoarthritis	Variable dosages across included trials (typically evaluated over weeks to months)	Chondroprotective potential, demonstrating that it helps prevent cartilage degradation and protects joint structures in osteoarthritis models	Inhibits the production of pro-inflammatory cytokines (IL-1β, TNF-α, IL-6), reduces cartilage-degrading enzymes (MMPs), and suppresses inflammatory signaling pathways like NF-κB	[163,164]

**Table 2:** Preclinical and Clinical Evidence for Combined Polyphenol and Conventional Pharmaceutical Therapy in Osteoarthritis Management.

Polyphenol	Combined Pharmaceutical	Study Type/Model	Molecular and Cellular Mechanisms	Key Findings and Clinical Outcomes	Reference
Epigallocatechin gallate (EGCG)	Hyaluronic acid/Chondroitin sulfate	Preclinical In Vitro/In Vivo (Osteoarthritis chondrocyte/joint degradation models)	Inhibits the NF-κB signaling pathway; reduces extracellular matrix (ECM) degradation; modulates bone turnover markers, promotes osteoblast differentiation, and suppresses osteoclastogenesis	Prevents articular cartilage matrix erosion; improves bone mineral density and bone microarchitecture, providing protective efficacy in bone loss and joint degeneration conditions	[185,186]
Resveratrol	Aspirin, Meloxicam	Preclinical In Vitro/In Vivo (Rat model of high-altitude NSAID injury) and Clinical (Pilot trial in knee osteoarthritis patients)	Directly targets and inhibits COX-2; modulates the TLR4/NF-κB/IκB pathway; restores gut microbiota balance; regulates serum type II collagen and aggrecan turnover markers	Prevents COX-2-mediated carcinogenesis; protects against NSAID-induced intestinal mucosal injury; improves articular matrix preservation and clinical status in knee osteoarthritis	[187,188,189]
Curcumin	Diclofenac	Clinical (Randomized controlled trial in patients with knee osteoarthritis) & Preclinical/Clinical Review (Systematic mechanistic insights in inflammatory complications)	Inhibits activation of NF-κB and AP-1 transcription factors; suppresses pro-inflammatory cytokines (IL-1β, TNF-α, IL-6); blocks the NLRP3 inflammasome signalosome; neutralizes reactive oxygen species (ROS)	Curcumin combined with diclofenac yields significantly greater improvements in pain relief and functional capacity with superior gastrointestinal tolerability compared to diclofenac monotherapy	[190,191,192]
Quercetin	Thermosensitive hydrogel drug delivery vehicle (evaluated for intra-articular co-administration with conventional local therapeutics)	Preclinical In Vivo (Rat model of osteoarthritis)	Suppresses pro-inflammatory cytokines (IL-1β, TNF-α, IL-6); downregulates matrix metalloproteinases (MMPs); inhibits cartilage matrix degradation pathways	Sustained intra-articular delivery attenuates cartilage erosion, reduces joint inflammation, and slows overall osteoarthritis progression	[193,194]
Luteolin	Indomethacin	Preclinical In Vivo (Rat model of indomethacin-induced gastric ulceration)	Inhibits pro-inflammatory cytokine pathways (TNF-α, IL-1β), reduces oxidative stress, suppresses COX-2/iNOS expression, and enhances gastric mucosal antioxidant defense mechanisms	Acts as a potent gastroprotective agent against indomethacin-induced mucosal damage, attenuates inflammatory responses, and offers potential as a natural adjunct to mitigate conventional NSAID toxicity	[195,196]
Genistein	Celecoxib (administered via a nanoliposomal formulation)	Preclinical In Vitro & In Vivo (Prostate cancer cell lines/models)	Inhibits the COX-2 pathway, downregulates Glucose Transporter-1 (Glut-1) receptors, suppresses NF-κB signaling, and reduces oxidative stress by scavenging reactive oxygen species (ROS)	Dual nanoliposomal co-delivery of genistein and celecoxib exhibits synergistic anti-proliferative and anti-inflammatory activity, significantly suppressing tumor cell growth and enhancing therapeutic efficacy while reducing systemic toxicity	[197,198]

**Table 3:** Comparative Overview of Real-World Pharmacological Interventions and Natural Polyphenols in Osteoarthritis Management.

Therapeutic Class	Representative Agents	Clinical Indication	Primary Mechanism of Action	Safety & Toxicity Profile	Treatment Persistence & Adjunctive Utility
Conventional NSAIDs	Aspirin, Ibuprofen, Diclofenac, Celecoxib, Meloxicam	Mainstay for acute flares and short-term pain relief	Analgesic & anti-inflammatory via COX inhibition	High toxicity risk with long-term exposure: • GI mucosal ulceration • Acute kidney injury • Hypertension & cardiovascular events	Low long-term persistence due to safety trade-offs and organ toxicities
Weak Opioids & Combination Regimens	Tramadol, Opioid/Analgesic combinations	Short- to mid-term options for pain management.	Central nervous system pain inhibition.	High risk of CNS side effects: • Nausea, dizziness, somnolence • Physical dependence • Increasing analgesic tolerance	Poor long-term persistence due to side effects, tolerance, and dependence
Natural Polyphenols	Curcumin, Resveratrol, EGCG, Quercetin, Luteolin, Genistein	Short-term symptomatic analgesia and long-term disease modification/adjunctive therapy	Modulates underlying inflammatory, oxidative, and catabolic cascades driving OA progression	Favourable systemic safety profile; minimal long-term systemic toxicity.	High treatment persistence; serves as an ‘analgesic-sparing’ agent (allows up to 50% NSAID dose reduction)

## Data Availability

No new data were created or analyzed in this study. Data sharing is not applicable to this article.
